# Glymphatic dysfunction in neuromyelitis optica spectrum disorder

**DOI:** 10.3389/fimmu.2025.1698986

**Published:** 2025-12-11

**Authors:** Ying Zhang, Hongxi Chen, Ziyan Shi, Rui Wang, Xiaofei Wang, Qin Du, Yuntao Mo, Shixiang Chen, Hongyu Zhou

**Affiliations:** 1 Department of Neurology, West China Hospital, Sichuan University, Chengdu, Sichuan, China; 2 General Practice Ward / International Medical Center Ward, General Practice Medical Center, West China Hospital, Sichuan University, Chengdu, Sichuan, China; 3 West China School of Medicine, Sichuan University, Chengdu, Sichuan, China

**Keywords:** glymphatic dysfunction, neuromyelitis optica spectrum disorder, DTI-ALPS, choroid plexus, perivascular spaces

## Abstract

**Background:**

Neuromyelitis optica spectrum disorder (NMOSD) involves aquaporin-4-mediated astrocyte injury, potentially impairing the glymphatic system. We assessed glymphatic function using the diffusion tensor image analysis along the perivascular space (DTI-ALPS) index, choroid plexus (CP) volume, and perivascular space (PVS) metrics, and explored associations with disability and brain structure.

**Methods:**

Thirty-nine aquaporin-4 immunoglobulin G (AQP4-IgG)-positive NMOSD patients without overt intracranial lesions and twenty-one age- and sex-matched healthy controls underwent 3T MRI using three-dimensional fast spoiled gradient-echo (3D-FSPGR) and diffusion tensor imaging (DTI). Manual segmentation of CP was performed in ITK-SNAP, and an automated pipeline derived DTI-ALPS and normalized PVS. Clinical assessments included the Expanded Disability Status Scale (EDSS), Hamilton Anxiety Rating Scale (HAMA) and Hamilton Depression Rating Scale (HAMD), Fatigue Impact Scale (FIS), and Pittsburgh Sleep Quality Index (PSQI). Statistical analyses comprised group comparisons, partial correlations (age/sex–adjusted), Firth penalized logistic regression and multivariable linear regression model, with false discovery rate (FDR) correction.

**Results:**

Among 39 NMOSD patients and 21 matched healthy controls, NMOSD patients showed trends toward higher DTI-ALPS (1.47 ± 0.10 vs. 1.45 ± 0.17, p = 0.20), larger CP volume (1,616 ± 408 mm^3^ vs. 1,600 ± 371 mm^3^, p = 0.80), and altered PVS (0.46 ± 0.06% vs. 0.45 ± 0.05%, p = 0.20). Within NMOSD, CP volume positively correlated with EDSS (r = 0.44, p = 0.002, FDR-corrected) and lateral ventricle volume (r = 0.46, p = 0.008, FDR-corrected). Baseline EDSS showed positive correlations with anxiety (HAMA; r = 0.36, p = 0.029) and depression (HAMD; r = 0.56, p < 0.001). In multivariable models, older age predicted lower odds of disability improvement (Coefficient = -0.024; 95% CI, -0.046 - 0.003; p = 0.029).

**Conclusions:**

In NMOSD, subtle CP volume enlargement is associated with disability status and ventricular enlargement, suggesting the presence of glymphatic dysfunction. CP alterations may represent a potential imaging biomarker of disease burden.

## Introduction

Neuromyelitis optica spectrum disorder (NMOSD) is an inflammatory disease of the central nervous system (CNS) characterized by pathogenic autoantibodies directed against aquaporin-4 (AQP4-IgG) (1). These antibodies gain access to the CNS either via endothelial transcytosis or through regions of blood–brain barrier (BBB) breakdown, where they bind AQP4 and activate the classical complement cascade, leading to infiltration of granulocytes, eosinophils, and lymphocytes, astrocyte injury, oligodendrocyte damage, demyelination, axonal loss, and neurodegeneration (2).

Recent work has identified a brain waste-clearance pathway termed the “glymphatic system” (3), in which CSF enters the parenchyma along periarterial spaces via astrocytic AQP4 channels, exchanges with interstitial fluid (ISF), and exits along perivenous spaces to eliminate metabolic byproducts (4). Glymphatic dysfunction has been implicated in multiple CNS inflammatory, demyelinating, and neurodegenerative disorders (5). Conventionally, human glymphatic function is assessed noninvasively by MRI quantification of perivascular spaces (PVS, or Virchow–Robin spaces) (6). More recently, Taoka et al. introduced diffusion tensor imaging analysis along the perivascular space (DTI-ALPS), which estimates glymphatic clearance by measuring water diffusivity in the white matter adjacent to the lateral ventricles (7, 8).

The CP-a key intracerebral structure responsible for CSF production and trafficking of proteins and immune cells-undergoes volume enlargement in various CNS inflammatory and neurodegenerative disorders, suggesting its involvement in brain inflammation and waste-clearance mechanisms (9). In multiple sclerosis (MS), CP enlargement correlates strongly with brain atrophy and clinical disability (10–12). Given mechanistic overlaps between NMOSD and MS, CP changes in NMOSD have attracted growing interest (13, 14), yet studies examining CP volume and its relationship to glymphatic dysfunction in NMOSD remain sparse.

Prior investigations have demonstrated marked glymphatic impairment in MS, linked to lesion burden, neurodegeneration, and disability (8, 15). Intriguingly, recent reports suggest NMOSD patients may exhibit comparable glymphatic disruption (16), potentially because AQP4 channels on astrocyte endfeet not only mediate NMOSD pathology but also drive CSF influx in the glymphatic pathway-hence, AQP4-IgG-mediated astrocyte injury could directly impair glymphatic clearance (17). However, specific studies of glymphatic function in NMOSD are lacking, and its role in disease progression and pathology remains undefined.

Accordingly, this study evaluates DTI-ALPS indices, CP volumes, and PVS metrics in NMOSD patients, and correlates these imaging markers with clinical disability, neuropsychiatric symptoms (anxiety, depression, fatigue, sleep disturbance), and structural brain damage, to elucidate glymphatic system involvement in NMOSD pathophysiology and inform future clinical management.

## Methods

### Study population

Between August 2016 and February 2022, we prospectively enrolled adults (≥18 years) with NMOSD from West China Hospital, Sichuan University, diagnosed according to the 2015 International Panel for NMO Diagnosis (IPND) criteria (1). Serum anti-AQP4-IgG was tested using a cell-based assay (CBA) on AQP4-transfected cells, with results interpreted according to laboratory cut-offs. We restricted inclusion to patients without overt intracranial lesions on conventional MRI to minimize confounding for analyses.

Exclusion criteria: (1) relapse or corticosteroid treatment within 3 months prior to assessment; (2) psychiatric disorders or other CNS diseases; (3) contraindications to MRI. Age- and sex-matched healthy controls (HCs) with normal neurological and MRI examinations were enrolled.

This study was approved by the Medical Ethics Committee of West China Hospital, Sichuan University, and all participants provided written informed consent.

### Clinical assessments

All subjects underwent demographic and neurological evaluations. Disability was rated by the Expanded Disability Status Scale (EDSS) (18); anxiety and depression by the Hamilton Anxiety Rating Scale (HAMA) (19) and Hamilton Depression Rating Scale (HAMD) (20); fatigue by the Fatigue Impact Scale (FIS) (21); and sleep quality by the Pittsburgh Sleep Quality Index (PSQI) (22). Trained raters administered all scales.

### MRI acquisition

Imaging was performed on a single 3T scanner (GE MR750) with a head fixation device. Subjects rested awake and eyes closed. Sequences acquired: (1) 3D T1-weighted structural (3D-FSPGR): TR = 5.16 ms; TE = 1.7 ms; TI = 450 ms; flip angle = 8°; FOV = 256 × 256 mm; matrix = 256 × 256; slice thickness = 1 mm; voxel = 1 × 1 × 1 mm; 192 slices. (2) Diffusion tensor imaging (DTI-SE-EPI): TR/TE = 8500/61.8 ms; flip angle = 90°; slice thickness = 2 mm; voxel = 2 × 2 × 2 mm; FOV = 256 × 256 mm; matrix = 128 × 128; 77 slices; b = 0,1000 s/mm²; 30 diffusion directions.

### Image processing and analysis

#### CP volume segmentation

**Figure 1:**
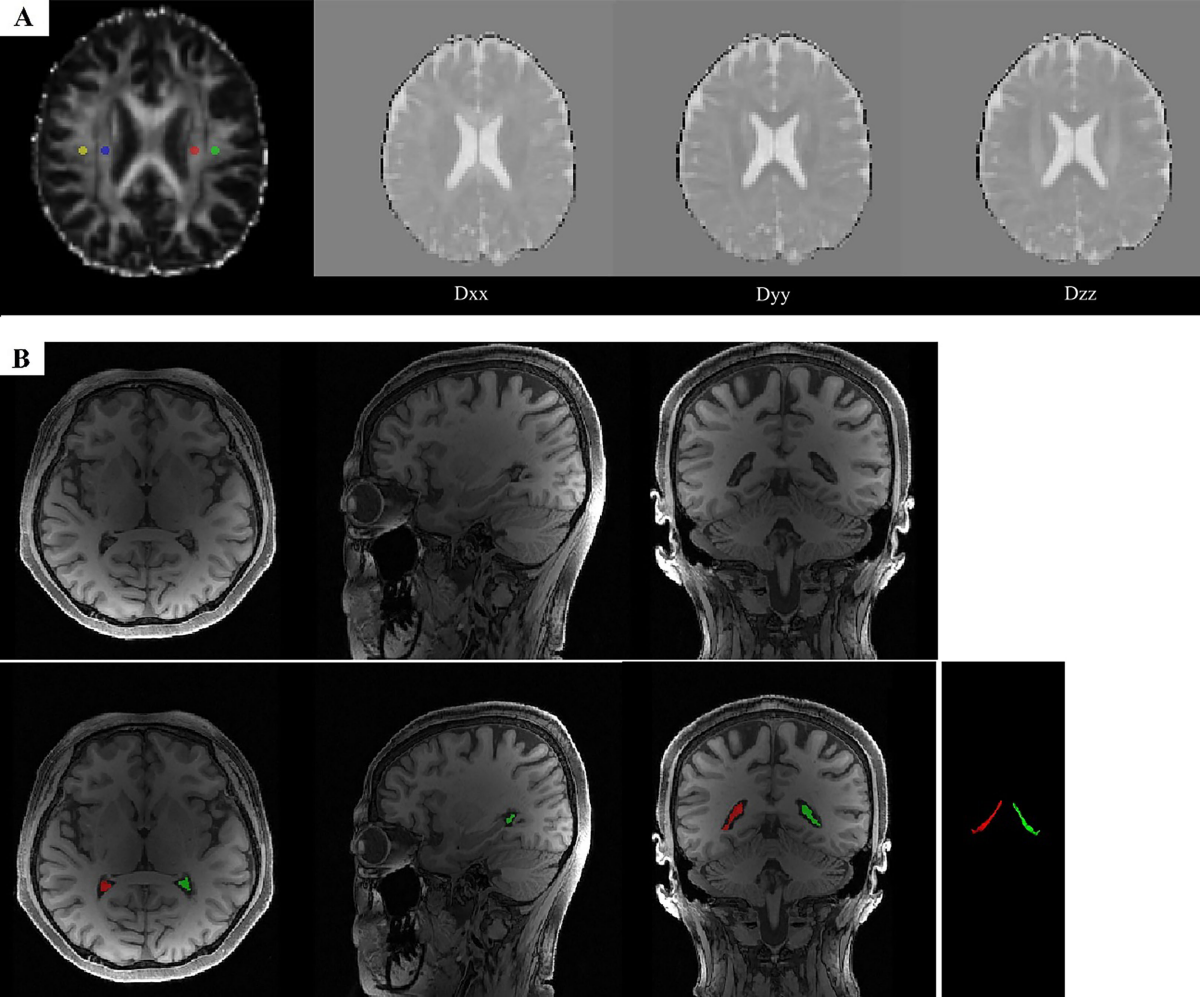
**(A)** Diffusion-weighted preprocessed images were used to calculate the DTI-ALPS index. Predefined ROIs for projection (superior corona radiata, yellow and green) and association (superior longitudinal fasciculus, blue and red) fibers at the level of the lateral ventricle body were used to derive the diffusivity values of Dxxproj, Dxxassoc, Dyyproj, and Dzzassoc. **(B)** Example of choroid plexus segmentation (before and after) on axial, sagittal, and coronal slices from a three-dimensional T1-weighted sequence.

The bilateral lateral ventricular CP was manually segmented on each subject’s 3D T1-weighted images in the sagittal, coronal, and axial planes using ITK-SNAP v4.2.0, by two experienced raters, who were blinded to clinical information and disease status (**Figure 1B**). Window width and level were adjusted as needed to delineate CP anatomical boundaries precisely. Volumes of the left and right CP were measured separately and summed to yield the total choroid plexus volume (CPV) (23). Inter-rater reliability was assessed using a random subset of 10 subjects. Intraclass correlation coefficients (ICC, two-way random-effects model, absolute agreement, single measures) with 95% confidence intervals were calculated, yielding ICC = 0.947 (95% CI: 0.797 - 0.987, p < 0.001) indicating excellent agreement. To account for inter-individual differences in head size, CP and other region-of-interest volumes were normalized by total intracranial volume.

#### DTI-ALPS index calculation

DTI data were processed using the FMRIB Software Library (FSL; University of Oxford). First, diffusion-weighted images (DWI) were used to compute per‐subject maps of fractional anisotropy (FA) and diffusion rates along the x, y, and z axes via the dtifit tool. Each subject’s FA map was then linearly registered to the Johns Hopkins University–International Consortium for Brain Mapping (JHU-ICBM) FA template using FSL’s flirt, and the resulting transformation matrix was applied to the three diffusivity maps. Next, guided by the JHU-ICBM-DTI-81 white-matter atlas, the superior corona radiata (SCR) and superior longitudinal fasciculus (SLF) at the level of the lateral ventricle body were identified (**Figure 1A**). Within each of these bilateral fiber bundles, 5 mm-diameter spherical regions of interest (ROIs) were automatically generated. To ensure anatomical accuracy, all ROIs were visually inspected and manually adjusted when necessary to match individual anatomy and avoid inclusion of CSF or gray matter. ROI placement was performed by a trained rater blinded to clinical information. Finally, the mean Dxx, Dyy, and Dzz values were extracted from the bilateral SCR and SLF ROIs, and the DTI-ALPS index was computed as previously described (24).

#### PVS segmentation

A fully automated pipeline was used to segment PVS (25). First, bias‐field correction (N4) was applied, and the denoised T1w and T2w images were registered to the 1 mm isotropic MNI‐152 standard space. Next, an Enhanced PVS Contrast (EPC) image was computed (EPC = T1w_denoise/T2w_denoise) to accentuate tubular fluid structures relative to surrounding tissue. In standard space, FSL‐FAST and FSL‐FIRST were employed to segment white matter (WM) and the subcortical nuclei (SubCor), respectively. Frangi vesselness filtering (tubularity > 0.5) was applied within WM and SubCor masks to extract candidate PVS, and 3D connected-component analysis removed clusters <5 voxels. Finally, PVS voxel volumes in WM, SubCor, and whole brain (WM + GM) were normalized to each region’s total volume and multiplied by 100% to yield percent PVS (pPVS).

### Statistical analysis

Data were analyzed in STATA 18 and G*Power 3.1. Normality was tested by Shapiro-Wilk. Normally distributed variables are presented as mean ± standard deviation; nonnormal as median (interquartile range); categorical as counts (percentages). Group comparisons used independent-samples t-tests or Mann–Whitney U tests for continuous variables, and chi‐square or Fisher’s exact tests for categorical variables. Partial correlations (controlling age and sex) examined associations between imaging and clinical measures. Relapse risk was modeled using Firth penalized logistic regression, while disability improvement was analyzed using multivariable linear regression. Multiple comparisons were corrected via false discovery rate (FDR). A sensitivity (*post-hoc*) power analysis was conducted to estimate the minimum detectable effect sizes for both between-group comparisons and within-cohort partial correlations, assuming two-tailed α = 0.05 and 80% power. All tests were two-tailed with α = 0.05.

## Results

### Demographics and clinical characteristics

A total of 39 NMOSD patients (mean age 44.92 ± 11.87 years; 37 females [94.9%]) and 21 HCs (mean age 44.30 ± 10.09 years; 20 females [95.2%]) were analyzed. Groups did not differ in age (p > 0.9), sex (p > 0.9), or education (p = 0.8). Among NMOSD patients, thirty-seven (94.9%) were AQP4-IgG seropositive. The remaining two seronegative patients fulfilled the 2015 IPND pathway for AQP4-IgG–negative/unknown NMOSD: each had ≥2 core clinical characteristics with dissemination in space-acute optic neuritis plus longitudinally extensive transverse myelitis (≥3 vertebral segments) on spinal MRI-no MS-typical brain MRI features, and alternative diagnoses excluded; MOG-IgG was negative where tested. The median baseline disease duration was 4.42 years (IQR: 2.12-8.79), and the median baseline EDSS was 3.50 (IQR: 2.00-4.00), which decreased to 2.75 (IQR: 1.00-4.00) at the last follow-up. During follow-up, 32 patients (82.1%) experienced at least one relapse. Key demographic and clinical data are summarized in **Table 1**.

**Table 1:** Demographic, clinical, and neuropsychological variables of the participants.

Characteristic	NMOSD, N = 39	HCs, N = 21	P value
Female,n (%)	37 (94.9)	20(95.2)	>0.9
Age, Mean ± SD	44.92 ± 11.87	44.30 ± 10.09	>0.9
Education years, Mean ± SD	9.52 ± 3.57	9.44 ± 3.72	0.8
AQP4-IgG seropositivity,n (%)	37 (94.9)	NA	
Baseline EDSS, median (IQR)	3.50 (2.00, 4.00)	NA	
Previous attacks, median (IQR)	4.00 (2.00, 5.50)	NA	
Baseline disease duration, median (IQR), years	4.42 (2.12, 8.79)	NA	
HAMA, median (IQR)	7.00 (3.50, 11.50)	NA	
HAMD, median (IQR)	7.00 (2.00, 13.50)	NA	
PSQI, median (IQR)	7.00 (4.00, 10.50)	NA	
Global FIS, median (IQR)	47.00 (23.00, 67.50)	NA	
Cognitive FIS, median (IQR)	8.00 (3.00, 16.50)	NA	
Physical FIS, median (IQR)	14.00 (16.50, 22.50)	NA	
Social FIS, median (IQR)	23.00 (11.50, 36.50)	NA	
Follow-up duration, median (IQR), years	8.04 (7.93, 8.17)	NA	
EDSS at last follow-up, median (IQR)	2.75 (1.00, 4.00)	NA	
ΔEDSS, median (IQR), %	0.00 (-21.97, 61.67)	NA	
Relapse during follow-up, n (%)	32 (82.1)	NA	

SD, standard deviation; IQR, interquartile range; EDSS, Expanded Disability Status Scale; HAMA, Hamilton Anxiety Rating Scale; HAMD, Hamilton Depression Rating Scale; PSQI, Pittsburgh Sleep Quality Index; FIS, Fatigue Impact Scale; ΔEDSS = (baseline EDSS-current EDSS)/baseline EDSS. P values were calculated using independent-sample t-test or Mann–Whitney U test for continuous variables, and Chi-square test or Fisher’s exact test for categorical variables. NA, not applicable.

### MRI comparisons

Compared with HCs, NMOSD patients showed slightly higher median DTI-ALPS index (1.47 ± 0.10 vs. 1.45 ± 0.17), larger CP volumes (1,616.03 ± 408.02 vs. 1,600.10 ± 370.73), and increased pPVS in the white matter (0.46 ± 0.06 vs. 0.45 ± 0.05), none reaching statistical significance (all p > 0.05). As shown in **Table 2**. *Post-hoc* power analysis indicated 80% power (α= 0.05, two-tailed) to detect a between-group difference of Cohen’s d = 0.77.

**Table 2:** Comparison of MRI features between NMOSD patients and HCs.

Characteristic	NMOSD, N = 39	HCs, N = 21	P value
ALPS, Mean ± SD	1.47 ± 0.10	1.45 ± 0.17	0.2
Left CP volume, Mean ± SD, mm^3^	741.41 ± 186.11	731.19 ± 188.61	>0.9
Right CP volume, Mean ± SD, mm^3^	874.62 ± 255.50	868.90 ± 225.01	>0.9
Total CP volume, Mean ± SD, mm^3^	1,616.03 ± 408.02	1,600.10 ± 370.73	>0.9
Total thalamus volume, Mean ± SD, mm^3^	12,635.84± 1,448.13	13,004.62± 1,293.72	0.3
Total lateral ventricle volume, Mean ± SD, mm^3^	15,573.01± 5,684.85	13,729.52± 4,291.18	0.2
Total intracranial volume, Mean ± SD, mm^3^	1,321,320.38± 101,238.65	1,337,949.87± 109,587.48	0.5
Normalized left CP volume, Mean ± SD, mm^3^	0.56 ± 0.15	0.55 ± 0.15	0.9
Normalized right CP volume, Mean ± SD, mm^3^	0.67 ± 0.20	0.65 ± 0.17	0.8
Normalized total CP volume, Mean ± SD, mm^3^	1.23 ± 0.33	1.20 ± 0.29	0.8
pPVS_in_WM, Mean ± SD	0.46 ± 0.06	0.45 ± 0.05	0.2
pPVS_in_SubCor, Mean ± SD	0.32 ± 0.05	0.34 ± 0.07	0.12
pPVS_total, Mean ± SD	0.78 ± 0.08	0.80 ± 0.07	0.7
Normalized total thalamus volume, Mean ± SD, mm^3^	9.57 ± 0.89	9.73 ± 0.72	0.6
Normalized total lateral ventricle volume, Mean ± SD, mm^3^	11.83 ± 4.31	10.24 ± 2.92	0.2

SD, standard deviation; DTI-ALPS, diffusion tensor imaging analysis along the perivascular space; CP, choroid plexus; pPVS, perivascular space; WM, white matter; SubCor, subcortical region. All p-values were FDR-adjusted.

### Correlations among MRI metrics

**Figure 2:**
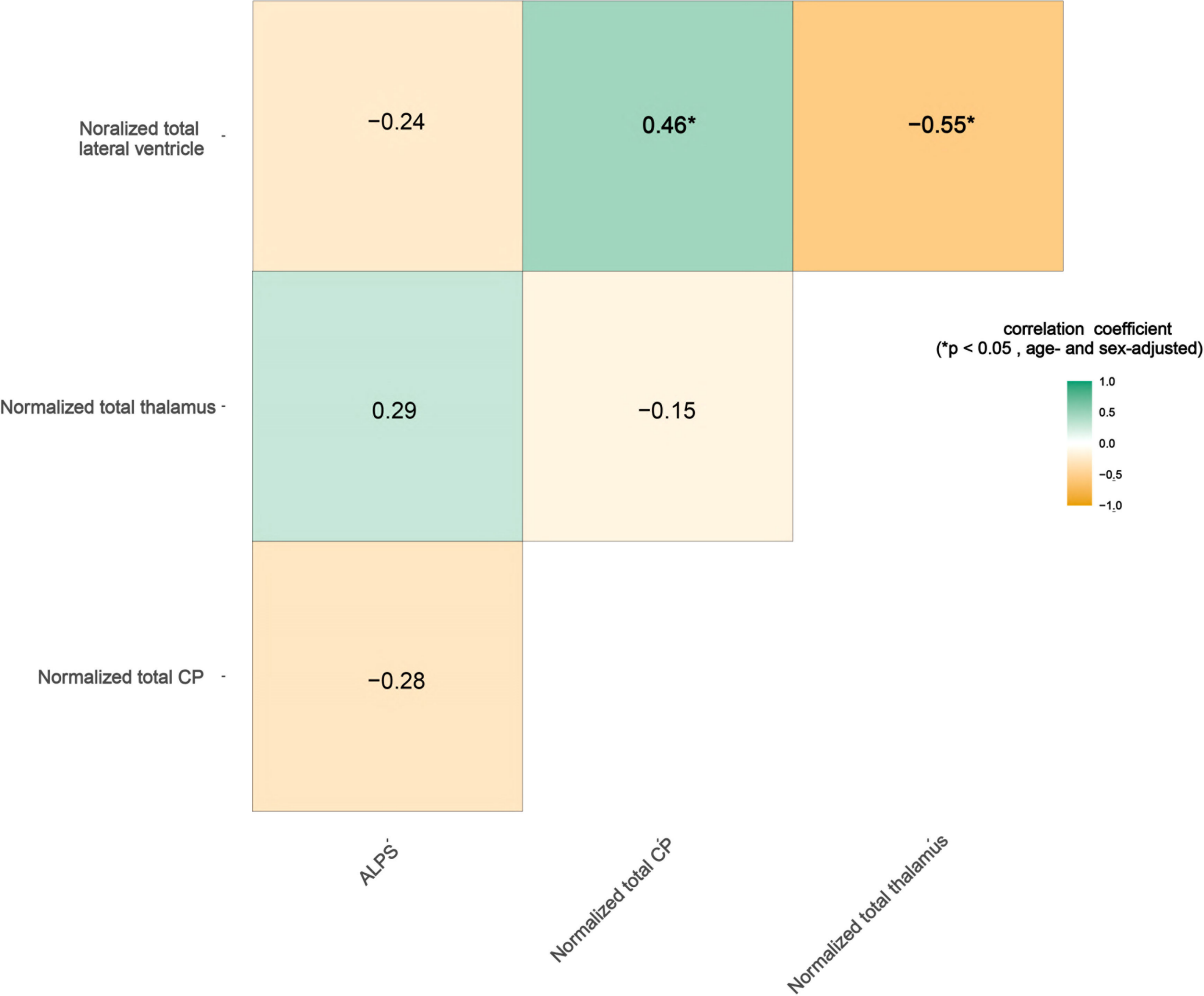
Correlations between the DTI-ALPS index and normalized total choroid plexus volume with conventional MRI measures. Numbers inside the boxes indicate the Spearman correlation coefficients. *p<0.05, indicating age- and sex-adjusted significantly.

After adjusting for age and sex, the normalized total CP volume in NMOSD patients correlated positively with normalized lateral-ventricle volume (r = 0.46, p = 0.008, FDR‐corrected). Conversely, normalized thalamic volume correlated negatively with normalized lateral-ventricle volume (r = -0.55, p = 0.023, FDR‐corrected), while CP and thalamic volumes were uncorrelated (p > 0.05). As shown in **Figure 2**.

### MRI–clinical associations

**Figure 3:**
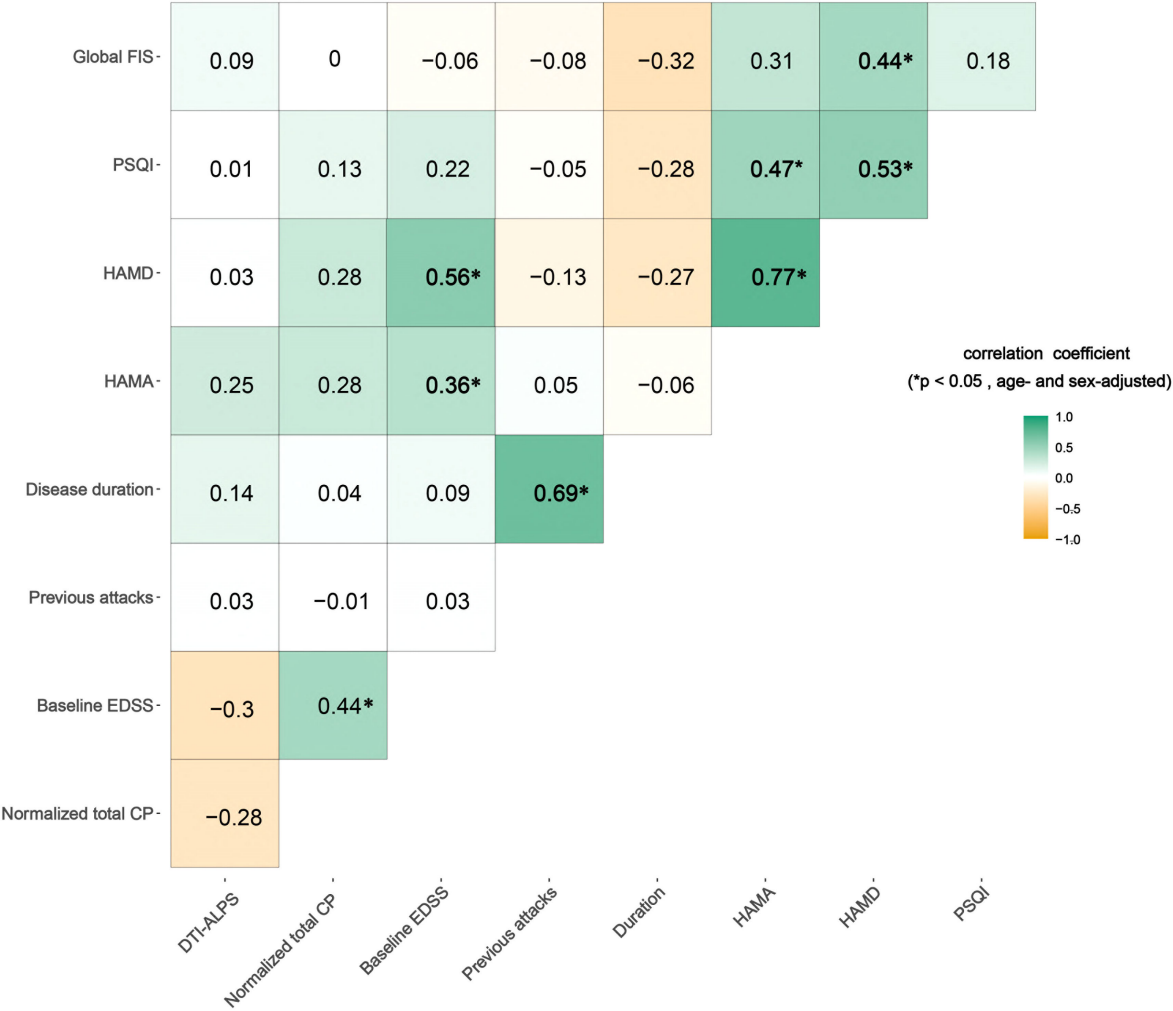
Correlations between the DTI-ALPS index and normalized total choroid plexus volume with clinical assessments. Numbers inside the boxes indicate the Spearman correlation coefficients. *p<0.05, indicating age- and sex-adjusted significantly.

In NMOSD patients, normalized total CP volumes correlated positively with baseline EDSS (r = 0.44, p = 0.002, FDR–corrected), indicating that greater disability was associated with larger CP volumes. Baseline EDSS also showed positive correlations with anxiety (HAMA; r = 0.36, p = 0.029) and depression (HAMD; r = 0.56, p < 0.001). Moreover, HAMA correlated strongly with HAMD (r = 0.77, p < 0.001) and with PSQI scores (r = 0.47, p = 0.004). HAMD likewise correlated with PSQI (r = 0.53, p = 0.001) and Global FIS scores (r = 0.44, p = 0.021). Finally, the number of disease relapses correlated with disease duration (r = 0.69, p < 0.001) (**Figure 3**). *Post-hoc* power analysis indicated 80% power (α= 0.05, two-tailed) to detect a within-group correlation of r = 0.43.

### Predictors of disability improvement

In a multivariable linear regression model adjusted for potential confounders, older age emerged as an independent predictor of a lower likelihood of disability improvement in NMOSD patients (Coefficient = -0.024; 95% CI, -0.046 - 0.003; p = 0.029), indicating that patients of greater age were less likely to experience improved disability during follow‐up. In a Firth penalized logistic regression with relapse as the outcome, no imaging or clinical covariates showed significant associations with relapse risk (all p > 0.05). Detailed estimates are provided in **Table 3**.

**Table 3:** Risk factors for relapse and improvement rate of disability during the median follow-up of 8 years.

Risk factors	Relapse^1^		Improvement rate of disability^2^ (ΔEDSS)	
	Coefficient (95% CI)	p-value	Coefficient (95% CI)	p-value
Age (per year)	-0.02(-0.08, 0.05)	0.621	-0.024(-0.046, -0.003)	0.029
Baseline EDSS	0.12(-0.32, 0.65)	0.607	0.125(-0.025, 0.275)	0.099
ALPS	1.22(-7.15, 9.83)	0.771	1.234(-1.635, 4.104)	0.387
Normalized total CP	-0.29(-3.27, 2.63)	0.841	0.286(-0.685, 1.256)	0.552

^1^Firth penalized logistic regression model

^2^Multivariable linear regression model.

ΔEDSS = (baseline EDSS-current EDSS)/baseline EDSS.

## Discussion

In this study, we investigated potential glymphatic dysfunction in NMOSD patients without overt intracranial lesions and its relationships with clinical disability, disease progression, and brain structural changes. Utilizing advanced MRI techniques-including the DTI-ALPS index, CPS, and PVS quantification-we characterized glymphatic function in this selected NMOSD cohort. Although we did not observe statistically significant group differences in glymphatic metrics between NMOSD patients and healthy controls, trends in CP enlargement and PVS alterations, together with their clinical correlations, suggest subtle glymphatic disturbances warranting further study.

CP enlargement has been widely reported in MS and other neuroinflammatory conditions and is thought to reflect chronic inflammation, blood–CSF barrier disruption, and neurodegeneration (10, 11, 26, 27). Data from NMOSD are conflicting: Chen et al. found no significant CP volume increase (11), whereas Kim et al. reported similar CP thickness but enhanced gadolinium uptake in NMOSD (13). Consistent with Müller et al. (14), our NMOSD cohort exhibited mildly increased CP volume compared with controls, which correlated positively with lateral ventricular volume. Moreover, CP volume was significantly associated with baseline EDSS, supporting CP enlargement as a potential imaging biomarker of clinical disability in NMOSD. Given the CP’s role in CSF production and immune surveillance-and its rich expression of AQP4 on the basolateral membranes of epithelial cells-it is plausible that pathogenic AQP4-IgG targeting of CP disrupts water channel function, leading to CP hypertrophy (28, 29). Future studies should integrate functional imaging of CP and correlate CP measures with inflammatory biomarkers or AQP4-IgG titers to clarify CP’s role in NMOSD immunopathology.

The DTI-ALPS index, a noninvasive proxy for glymphatic function, has been validated in various neurological disorders (30, 31). In MS and MOG-IgG-associated disorders, reduced ALPS indices correlate with disease severity and brain atrophy (8, 15, 32). In contrast, our NMOSD patients demonstrated a non-significant, slightly elevated DTI-ALPS compared with controls, diverging from prior reports of ALPS reduction in NMOSD (16, 33). This discrepancy likely stems from our rigorous exclusion of patients with overt intracranial lesions to capture early, subclinical glymphatic changes; however, this criterion may have limited detectable alterations below the statistical threshold. Additionally, glymphatic impairment in MS is closely tied to lesion burden and atrophy (8, 15), whereas NMOSD pathology predominantly affects AQP4-rich optic nerves and spinal cord, sparing brain parenchyma, which may explain the preserved ALPS indices. It also further suggests that glymphatic dysfunction in NMOSD may depend on lesion location and severity.

PVS enlargement is another hallmark of glymphatic impairment. We observed region-specific trends: a slight increase in white matter PVS volume but modestly reduced subcortical and global PVS volumes in NMOSD compared with controls. These patterns mirror Cacciaguerra et al.’s findings of elevated centrum semiovale PVS scores but unchanged basal ganglia scores in NMOSD (33). Lack of significant PVS expansion in our cohort may reflect early disease stage and absence of macroscopic lesions, with pathological PVS enlargement not yet exceeding detection thresholds. Future work should include NMOSD patients across clinical stages and varying lesion burdens to fully elucidate PVS dynamics.

Additionally, in our longitudinal follow‐up of NMOSD patients, measures of glymphatic dysfunction did not independently predict disability recovery. Instead, multivariable logistic regression identified older age as the sole independent predictor of reduced likelihood of disability improvement-a finding in line with observations across neuroinflammatory conditions, where advancing age consistently portends poorer recovery and outcomes (34). Aging may disrupt AQP4 polarization at astrocytic endfeet, impairing CSF-ISF exchange and thereby promoting neurodegenerative processes (35, 36). Concurrently, immunosenescence-characterized by diminished microglial phagocytic capacity, skewing of T-cell subsets, and reduced regulatory T-cell generation-may hinder the resolution of CNS inflammation and perpetuate tissue injury in older individuals (37). Although our study focused on surrogate markers of perivascular clearance such as the DTI-ALPS index and PVS volume, these age‐dependent mechanisms likely underlie the attenuated functional recovery we observed, independent of measurable changes in perivascular clearance metrics. Clinically, these insights underscore the imperative for early and intensive intervention in elderly NMOSD patients, targeting not only astrocyte and myelin injury but also the preservation or enhancement of perivascular clearance pathways that may be secondarily compromised by aging.

Several limitations merit consideration. First, the relatively modest sample size may have limited the ability to detect small glymphatic alterations, even though the study was adequately powered for moderate-to-large effects. Future studies with larger cohorts are warranted to validate these findings. Second, excluding patients with visible intracranial lesions reduced potential confounding from lesion-related structural changes but may have biased the sample toward milder disease and limited generalizability to the broader NMOSD population. Third, DTI-ALPS remains an indirect measure of CSF-ISF exchange and cannot fully capture true glymphatic flow; direct tracking methods (e.g., dynamic contrast-enhanced MRI or PET) will be needed to validate and extend our findings.

## Conclusions

In NMOSD patients without overt intracranial lesions, we observed subtle CP enlargement and region‐specific PVS alterations, with CP volume correlating positively with disability and ventricular size. Although DTI-ALPS and normalized PVS metrics did not show significant changes, our findings highlight CP enlargement as a potential imaging biomarker of subclinical neuroinflammation and disease burden in NMOSD. Future studies with larger, more heterogeneous cohorts and direct glymphatic imaging approaches are warranted to clarify the contribution of glymphatic dysfunction to NMOSD pathophysiology and to evaluate its potential as a therapeutic target.

## Data Availability

The raw data supporting the conclusions of this article will be made available by the authors, without undue reservation.
